# Overview of Brain-to-Gut Axis Exposed to Chronic CNS Bacterial Infection(s) and a Predictive Urinary Metabolic Profile of a Brain Infected by *Mycobacterium tuberculosis*

**DOI:** 10.3389/fnins.2020.00296

**Published:** 2020-04-21

**Authors:** Simon Isaiah, Du Toit Loots, Regan Solomons, Martijn van der Kuip, A. Marceline Tutu Van Furth, Shayne Mason

**Affiliations:** ^1^Human Metabolomics, Faculty of Natural and Agricultural Sciences, North-West University, Potchefstroom, South Africa; ^2^Department of Pediatrics and Child Health, Faculty of Medicine and Health Sciences, Stellenbosch University, Tygerberg, South Africa; ^3^Pediatric Infectious Diseases and Immunology, Amsterdam University Medical Center, Academic Medical Center, Emma Children’s Hospital, Amsterdam, Netherlands

**Keywords:** gut-brain axis, tuberculous meningitis, immunological biomarker, metabolism, urinary profiling, chronic neuroinflammation, bacterial infectious diseases

## Abstract

A new paradigm in neuroscience has recently emerged – the brain–gut axis (BGA). The contemporary focus in this paradigm has been gut → brain (“bottom-up”), in which the gut-microbiome, and its perturbations, affects one’s psychological state-of-mind and behavior, and is pivotal in neurodegenerative disorders. The emerging brain → gut (“top-down”) concept, the subject of this review, proposes that dysfunctional brain health can alter the gut-microbiome. Feedback of this alternative bidirectional highway subsequently aggravates the neurological pathology. This paradigm shift, however, focuses upon non-communicable neurological diseases (progressive neuroinflammation). What of infectious diseases, in which pathogenic bacteria penetrate the blood–brain barrier and interact with the brain, and what is this effect on the BGA in bacterial infection(s) that cause chronic neuroinflammation? Persistent immune activity in the CNS due to chronic neuroinflammation can lead to irreversible neurodegeneration and neuronal death. The properties of cerebrospinal fluid (CSF), such as immunological markers, are used to diagnose brain disorders. But what of metabolic markers for such purposes? If a BGA exists, then chronic CNS bacterial infection(s) should theoretically be reflected in the urine. The premise here is that chronic CNS bacterial infection(s) will affect the gut-microbiome and that perturbed metabolism in both the CNS and gut will release metabolites into the blood that are filtered (kidneys) and excreted in the urine. Here we assess the literature on the effects of chronic neuroinflammatory diseases on the gut-microbiome caused by bacterial infection(s) of the CNS, in the context of information attained via metabolomics-based studies of urine. Furthermore, we take a severe chronic neuroinflammatory infectious disease – tuberculous meningitis (TBM), caused by *Mycobacterium tuberculosis*, and examine three previously validated CSF immunological biomarkers – vascular endothelial growth factor, interferon-gamma and myeloperoxidase – in terms of the expected changes in normal brain metabolism. We then model the downstream metabolic effects expected, predicting pivotal altered metabolic pathways that would be reflected in the urinary profiles of TBM subjects. Our cascading metabolic model should be adjustable to account for other types of CNS bacterial infection(s) associated with chronic neuroinflammation, typically prevalent, and difficult to distinguish from TBM, in the resource-constrained settings of poor communities.

## Introduction

A new paradigm in neuroscience has emerged in recent years – the brain–gut axis (BGA) – involving bidirectional communication between the brain and gut. This implicates a variety of pathways, including the enteric nervous system (ENS), central nervous system (CNS), gastrointestinal tract (GIT), endocrine system/GI hormones, and immune response, all integrated to orchestrate the bidirectional feedback loop of the BGA. As averred by Hippocrates, the Greek physician acknowledged by many as the father of modern medicine, “*All disease starts in the gut*.*”* The gut-microbiome is made up of innumerable microbes, which function in a mutualistic relationship with the human host (Collins et al., 2012; Zhu et al., 2017). Currently, scientific evidence supports the notion that homeostatic imbalance is initiated in the gut-microbiome, mediated by several microbe-derived molecules, in the gut–brain (“bottom-up”) direction of communication (Foster and Neufeld, 2013; Martin et al., 2018). Stable gut microbiota are essential for normal gut physiology and contribute to appropriate signaling along the BGA (Forsythe et al., 2010; Cryan and Dinan, 2012; Schroeder and Bäckhed, 2016). Over the past decade, however, neuroscience research on the BGA has focused on how perturbations in the gut-microbiome affect the brain in a feedback loop, centered on the premise of “*you are what you eat”* and “*gut feelings”* (Moos et al., 2016; Sherwin et al., 2016; Zmora et al., 2019). Considering the bottom-up motif, particularly its perturbations in the gut-microbiome, can have a clear and direct effect on the host’s psychological state-of-mind (depression, anxiety, bipolar disorder), behavior (autism) and also in the pathogenesis and/or progression of various neurodegenerative diseases (Alzheimer’s, Parkinson’s, and multiple sclerosis). These disorders associated with the bottom-up direction of communication have been succinctly and meticulously detailed in many topical research reviews (Mayer et al., 2014; Konturek et al., 2015; Powell et al., 2017; Zhu et al., 2017; Martin et al., 2018; Ambrosini et al., 2019). Perturbations of the BGA associated with non-communicable neurological diseases – to what degree, the precise mechanism involved, and their appropriate therapy – are not yet well understood. Many studies on the role of microbiota in the pathogenesis of neurodegenerative/psychiatric diseases exist, however, and their main findings are summarized in Table 1.

**TABLE 1:** Main findings from studies describing the role of microbiota in the pathogenesis of neurodegenerative/psychiatric diseases.

**Disorders**	**Main findings**	**References**
**Neurodegenerative**		
Parkinson’s disease (PD)	(i) Gut microbiota influence the activity of enteric neurons, affecting cellular α-synuclein (α-syn) secretion, characterized by the accumulation and aggregation of α-syn in the substantia nigra (SN).	Braak et al., 2003
	(ii) Gastrointestinal dysfunction is present in ∼80% of PD patients.	Mulak and Bonaz, 2015
	(iii) α-Synucleinopathy is suggested to be an early indicator of PD pathology.	Nair et al., 2018
	(iv) The vagal nerve, which serves as channel for α-syn from the ENS to the CNS, is crucial for the communication between gut microbiota and the brain.	Ulusoy et al., 2013; Scheperjans et al., 2015; Fitzgerald et al., 2019
	(v) Pathological hallmarks of PD are a loss of dopaminergic neurons in the SN and the presence of cytoplasmic eosinophilic inclusions termed Lewy bodies (LBs).	Lebouvier et al., 2009
	(vi) Immunolabeling with α-syn antibodies have become the reference standard in the assessment of LBs and Lewy neurites in both the CNS and peripheral nervous system. Hence, α-synucleinopathy affects all levels of the BGA.	Lebouvier et al., 2009
Alzheimer’s disease (AD)	(i) AD is characterized by a deposition of amyloid beta (Aβ) followed by the formation of plaques, characterized by a progressive decline in cognitive function.	Wang et al., 2014; Jouanne et al., 2017
	(ii) Gut microbiota produce amyloids which aid bacterial cell binding, and form part of the biofilm protecting these from destruction by host immune factors.	Friedland and Chapman, 2017
	(iii) Bacterial amyloid proteins exposure to the host, from the gut, may be detrimental since they prime of the host’s immune system against endogenous production of neuronal amyloids in the brain.	Kowalski and Mulak, 2019
	(iv) Bacterial lipopolysaccharides are increased in the neocortex and the hippocampus in AD.	Zhao et al., 2017
	(v) Calprotectin is indicative of inflammation and has be detected in elevated amounts in the CSF, brain and fecal matter of AD patients.	Kowalski and Mulak, 2019
Multiple sclerosis (MS)	(i) MS is a demyelinating disease, clinically associated with autoimmune disease. Progressive degradation of the integrity of the epithelia that comprise cellular barriers essential to maintaining the integrity of both intestine and CNS, have been associated in MS patients suffering from autoimmunity, resulting in paralysis and other related symptoms of MS.	Ochoa-Repáraz and Kasper, 2014; Dendrou et al., 2015; Ochoa-Repáraz et al., 2018
	(ii) Clinical signs of MS are relapse of sensory, motor and cerebellar complications; while an acute disease stage is a characteristic feature of the relapsing-remitting MS (the latter of which are often diagnosed with neuronal dysfunction).	Johnston and Joy, 2001; D’Amico et al., 2016; Connick et al., 2018
	(iii) Secondary-progressive MS develops and transcends into progressive neurological impairment.	D’Amico et al., 2016
	(iv) Dysbiosis affects the immunological responses of the host to the microbiota, as described in an experiment where germ-free mice with an immune dysfunction, were characterized by an imbalance between pro- and anti-inflammatory immune cells in the gut, where after colonization of the gut with commensal microbes restored immune function.	Mazmanian et al., 2005; Kirby and Ochoa-Repáraz, 2018; Ochoa-Repáraz et al., 2018
**Neuropsychiatric**		
Autism spectrum disorders (ASD)	(i) Dysbiosis in children with ASD has been show to contribute to both gastrointestinal and CNS abnormalities.	Wang et al., 2011; Santocchi et al., 2016
	(ii) Short-chain fatty acid producing bacteria, and their metabolites, especially propionic acid, has been indicated to adversely affect the CNS and contribute to autism behavior by modulating the BGA.	De Angelis et al., 2015
	(iii) Behavioral abnormalities are accompanied by imaging abnormalities in the sensory and emotion regulation regions of the brain.	Green et al., 2013
	(iv) Abnormally elevated levels of lipopolysaccharides have also been associated with the pathogenesis of autism.	Fattorusso et al., 2019
	(v) 40% of ASD patients complain of GI symptoms; abnormalities such as chronic diarrhea, constipation, vomiting, feeding problems, reflux and abdominal pain, as well as anxiety.	Mayer et al., 2014; Fattorusso et al., 2019
	(vi) Patients with ASD also have high fecal and urinary levels of bacterially derived p-cresol, and further exposure to p-cresol has been shown to contribute to the severity of behavioral symptoms and cognitive impairment in ASD.	Altieri et al., 2011; Persico and Napolioni, 2013; Gabriele et al., 2014
	(vii) Optimized remedies that are practiced include rehabilitation, educational therapy and psycho-pharmacological approaches.	Fattorusso et al., 2019
Depression, anxiety, and major depressive disorder (MDD)	(i) Pre-clinical studies of depression, anxiety and MDD indicate that the altered brain function associated with these, can partly be attributed to disturbances in the gut microbiota composition.	Bercik et al., 2011; Park et al., 2013; Jiang et al., 2015; Kelly et al., 2016
	(ii) Studies have shown that the microbiome has the capacity to influence on emotional behavior, and is associated with various parameters relating to depression pathogenesis and severity.	Bercik et al., 2011; Clemente et al., 2012; Cryan and Dinan, 2012
	(iii) Hippurate, dimethylamine and dimethylglycine, all by-products of gut microbiota, have been detected in abnormal concentrations in MDD patients which further substantiates the aforementioned observations.	Zheng et al., 2013, 2016
	(iv) Increased severity in depression and anxiety have been noted following bacterial infection in patients.	Naseribafrouei et al., 2014

The focus of this review is on the brain–gut (“top-down”) direction of the BGA. In particular, perturbations of brain metabolism induced by invading bacteria and, as a consequence, gut dysbiosis. Within the contemporary paradigm of a perturbed BGA, most of the relevant research centers on non-communicable neurological diseases, synonymous with a slow, gradual progression of neuroinflammation. However, the link between the brain–gut concept and CNS bacterial infection(s) is less prevalent in the literature, and hence the focus of this review. The most recent and comprehensive review of the BGA was by Cryan et al. (2019). However, only a very small section, amounting to half a page, discusses infections and the brain, even though bacterial penetration of the blood–brain barrier (BBB), and subsequent infection, leads to a cascade of events within the brain, modulating a feedback effect on the host gut-microbiome (Dando et al., 2014; Bauer et al., 2016; Martin et al., 2018). Bacterial infection(s) of the CNS induce an inflammatory response via glia mediators, pivotal to establishing communication between the host’s immune system and the brain (DiSabato et al., 2016) and, ultimately, generating sustained feedback on the BGA (Geyer et al., 2019).

As a proof of a novel concept for the BGA, we use three previously validated immunological CSF markers of tuberculous meningitis (TBM) – vascular endothelial growth factor (VEGF), interferon-gamma (IFN-γ), and myeloperoxidase (MPO) – to model/predict the metabolic changes, and are the basis for postulating a metabolic cascade, expected within the brain of a TBM patient. It is well known that important diagnostic and prognostic information related to alterations in metabolic cascades and disruption of homeostasis can be characterized through metabolite profiling of urine (An and Gao, 2015; Emwas et al., 2015). Hence, logic dictates that if the BGA exists then the impact of chronic CNS bacterial infection(s) (such as TBM) should be reflected in the host’s urine.

## Brain–Gut Concept

According to the brain–gut (“top-down”) concept, the brain can alter the community structure and function of the gut-microbiome in a bidirectional interaction feedback loop, characterized by continuous communication between the CNS and the GIT (Zhu et al., 2017; Karol and Agata, 2019). The GIT is a highly complex organ involved in multiple dynamic physiological processes, while interacting with the gut-microbiome – an extensive and diverse community of bacteria (Parker et al., 2018). The brain nerves (e.g., vagus nerve), which control unconscious tasks, run from the brainstem to the gut, maintaining the physical bidirectional communication between the CNS and intestinal wall. The brain-to-gut signaling pathway affects host–bacteria interactions in the GIT by influencing the enteric microbiota indirectly via an altered intestinal permeability, or directly via signaling molecules released into the gut lumen from immune and enterochromaffin cells, thereby increasing motor, sensory and secretory modalities of the GIT (Rhee et al., 2009; Grenham et al., 2011; Eisenstein, 2016). Those signaling systems that allow the brain, in this crosstalk communication, to influence gut-microbiome functions in the GIT, are: (1) the endocrine-immune system, (2) the hypothalamus–pituitary–adrenal (HPA) axis, (3) the sympathetic and parasympathetic arms of the autonomic nervous system (ANS), and (4) enteric nervous system (ENS) (Rhee et al., 2009; Grenham et al., 2011; Cong et al., 2015). These signaling systems are interlinked systematically to form a complex reflex network, with afferent and efferent fibers (O’Mahony et al., 2011). Hence, activation of any of these signaling systems, either alone or in combination, might influence the composition and functionality of enteric microbiota (Rhee et al., 2009). For instance, under conditions of chronic stress the brain recruits these same mechanisms, by activation of the HPA axis in the brain, to regulate cortisol secretion. Cortisol in turn affects various immune cells (including cytokine secretion) locally in the gut, subsequently inducing changes to microbiota composition, and increasing the gastrointestinal permeability (de Punder and Pruimboom, 2015; Kelly et al., 2015; Farzi et al., 2018). Hence, an exceedingly complex array of signaling systems, all interlinked, lies between the brain and gut in the “top-down” concept (Aziz and Thompson, 1998; Collins and Bercik, 2009; O’Mahony et al., 2009; Forsythe et al., 2014; Khlevner et al., 2018; Weltens et al., 2018; Zhao et al., 2018).

The CNS is well shielded by the BBB, the major site of blood–CNS exchange. The barrier comprises microvascular endothelial cells, astrocytes and pericytes, and is tasked with the regulated passage of molecules into and out of the brain (Abbott et al., 2010; Sochocka et al., 2017b). Neurotropic bacteria are capable of evading host defenses, gaining access to the CNS (Dando et al., 2014), with >95% of brain abscesses caused by bacterial infection(s) (Sonneville et al., 2017). Furthermore, the brain may become particularly susceptible to bacterial infection(s), if the BBB is chronically compromised by an initial infection (Mendes et al., 1980; Cantiera et al., 2019). Various brain cells – microglia (resident macrophages), endothelial, ependymal, neuronal and glial (astrocytes and oligodendrocytes) – convey innate immune molecules that prompt the recruitment of leukocytes into the infected CNS compartments, in order to combat invading neurotropic bacteria (Klein et al., 2017). This process results in a series of initial neuroinflammatory events within the brain, as well as phagocytosis of the infecting bacteria, in an attempt to control disease progression. Neuroinflammation in the CNS is mediated by the production of cytokines and chemokines, that are pivotal in the coordinated communication between the immune system and the brain (DiSabato et al., 2016). The host’s inflammatory reaction in the CNS is initiated by the recognition of the invading pathogens, which in turn leads to the local production of mediators by the glial cells comprising microglia and astrocytes (Grandgirard et al., 2013). Thus, acute inflammatory feedback is triggered by rapid and early activation of mediators released by activated glial cells in the CNS due to the infectious agent. However, when the presence of an infectious agent persists, a chronic state of inflammation within the brain results (Sochocka et al., 2017a) and the activated glial cells are altered beyond “normal” proportions, which results in progressive neurodegeneration (Kempuraj et al., 2017; Sochocka et al., 2017a). Pattern recognition receptor (Newton and Dixit, 2012; Suresh and Mosser, 2013) activation initiates the release of pro-inflammatory cytokines and chemokines, in order to modulate the immune response, leading to pleocytosis of white blood cells (Janowski and Newland, 2017). This in turn triggers an increased BBB permeability and the influx of leukocytes from the blood into the CNS at the site(s) of infection (Waisman et al., 2015; Kempuraj et al., 2017). Although this is the mechanism by which the brain attempts to restore homeostasis and protect itself against the invading pathogen (More et al., 2013), the chronic production of immune cells induces neurodegeneration. Since activated microglia have both neuroprotective and neurotoxic functions (Kim, 2003; Nimmerjahn et al., 2005; Dando et al., 2014; Liechti et al., 2015; Doran et al., 2016), various toxic molecules released by the microglia during the immune response may also inflict neuronal injury.

## Bacterial Infections of the CNS and Their Effect on the Brain–Gut Axis

Most bacterial CNS infections present acutely, including subacute and chronic forms. Common acute bacterial CNS infections involve *Streptococcus agalactiae*, Gram-negative bacilli including *Escherichia coli, Klebsiella pneumoniae*, *Listeria monocytogenes, Neisseria meningitidis*, and *Streptococcus pneumoniae* (Durand et al., 1993; Gray, 1997; Grandgirard et al., 2013; Zhou, 2019), while subacute and chronic bacterial CNS infections, besides *Mycobacterium tuberculosis*, involve *Borrelia burgdorferi*, *Leptospira interrogans*, *Treponema pallidum, Mycobacterium leprae*. Microbial pathogens can gain entry into CNS by penetrating the BBB or via the olfactory (Kristensson, 2011). The nasopharynx is the usual portal of entry for major meningeal pathogens. Pathogens penetrate the olfactory epithelium, and could potentially cross epithelial barriers into the subarachnoid space; compromising the epithelial tissue by exposure to bacterial virulence factors, directly infecting the olfactory sensory neurons (Dando et al., 2014; Rey et al., 2018). Meningeal invasion subsequently follows via penetration of the cellular barriers of the CNS. The putative cascade of events caused by bacterial infection(s) of the brain that alter permeability of the gut – discussed in detail below, ultimately leads to dysbiosis.

(1) Within the cascade, the first step of bacterial invasion involves transitioning across the compromised BBB into the subarachnoid space. Pathogens can cause disruption of the BBB, which enables their passage into the brain. The various host defenses are usually inadequate to control the infection. Leukocytes traverse the BBB and patrol the brain parenchyma under normal conditions. During inflammation, as result of infection, the BBB junctions (adherens and tight) that regulate the flux of ions, polar molecules, and macromolecules from the systemic circulation can be compromised, thus traffic is greatly increased at these junctions. Bacteria may cross the BBB by transcellular penetration after bacterial adhesion to endothelial cells or via infected leukocytes. Pinocytosis, increased by leukocytes combating bacteria that might have invaded following disruption of tight junctions or via the “Trojan horse” mechanism – phagocytes infected with the pathogen transverse the BBB (Kim, 2003; Pulzova et al., 2009). Leukocytes, activated by inflammatory molecules released during infection, cross the BBB by a multistep process that involves attachment to, and invasion through, the post-capillary venule wall and the surrounding endothelial and parenchymal basement membranes which differ in their laminin composition and permeability (Owens et al., 2008; Kristensson, 2011; Dando et al., 2014). During infection of the CNS various acute pathological events may occur which further compromise the CNS. The brain parenchyma is populated by resident immune cells, the microglia, which are highly specialized tissue macrophages.
(2) Microglia cells, the primary immune effector cells in the brain, continuously survey the brain parenchyma and respond to very subtle alterations in their microenvironment and in the brain’s structural integrity (Nimmerjahn et al., 2005). Microglia are highly motile immune effector cells in the brain that respond to neuronal infection and damage. The role of microglia in a healthy brain, along with immediate reaction to brain damage, is paramount in response to the prevention of any kind of major brain damage. Microglia are considered essential for communication in the intrinsic immune system of the CNS, as well for intercellular crosstalk between astrocytes and neurons (Kreutzberg, 1996; Stollg and Jander, 1999; Streit, 2002; Streit et al., 2004; Akiyoshi et al., 2018). Microglia maintain CNS health via mediators involved in the function of neurogenesis, modeling of synapses, excitotoxicity prevention and regulation of neuroinflammation. Short-chain fatty acids derived from the gut-microbiome play a pivotal role in the function and maturation of microglia. Hence, microglia are crucial mediators in the interaction between the CNS and the gut microbiota (Wang et al., 2018; Abdel-Haq et al., 2019).
(3) Bacterial cell wall material, enzymes, and toxins cause direct injury to neurons and indirect damage by increasing vascular permeability that causes edema and further injury. Microglial cells respond to bacterial pathogens and neuronal injury by the production of reactive oxygen species (ROS), nitrous oxide, and peroxynitrite. Immune response also contribute to neurotoxicity via release of proteases and excitatory amino acids. Several signaling molecules, such as catecholamines, serotonin, dynorphin and cytokines, used by the host for neuronal and neuroendocrine signaling, are also likely to be secreted into the gut lumen (Rhee et al., 2009).
(4) Bacterial pathogens may target neurons and glial cells, inducing inflammation and exerting direct cytopathic effect due to the release of their products. Thereafter, brain cell apoptosis begins to occur. For example, Pneumolysin and hydrogen peroxide (H_2_O_2_) are direct triggers of *Streptococcus pneumoniae*. H_2_O_2_ rapidly diffuses through eukaryotic cell membranes to damage intracellular targets thus increasing intracellular Ca^2+^, damaging mitochondria, and causing the release and translocation of mitochondrial apoptosis-inducing factor. Increased intracellular ROS and Ca^2+^ precedes morphologic changes that lead to brain cell apoptosis (Mitchell and Andrew, 1997; Lipton and Nicotera, 1998; Braun et al., 2002; Janowski and Newland, 2017). Brain cell apoptosis leads to neuronal injury in the form of brain manifestations, such as: basal ganglia and thalami communication that become obstructive, cranial nerve dysfunction, minor focal neurological signs, infiltrates of inflammatory cells, exudation of protein-rich fluid, and edema (Gray, 1997; Hussein and Shafran, 2000; Van de Beek et al., 2004; Østergaard et al., 2005; Al Khorasani and Banajeh, 2006; Hähnel and Bendszus, 2009; Abdulrab et al., 2010).
(5) Pathogenic bacteria that causes meningitis exhibit antiphagocytic capsular polysaccharide ability which enables survival within the blood. Hence, changes in the gut involves hematogenous dissemination of bacteria, initiating meningitis via mucosal adhesion of the organism and subsequent systemic invasion (Seib et al., 2009; Harvey et al., 2011; Dando et al., 2014). The intestinal immune system is tasked to maintain homeostasis within the gut-microbiome via the processes of minimizing direct contact between intestinal bacteria and the epithelial cell surface (stratification), and confining penetrant bacteria to intestinal sites and limiting their exposure to the systemic immune compartment (compartmentalization) (Hooper et al., 2012; Macpherson and McCoy, 2013). Mucosal surfaces represent the major interface and constitute the point of entry of most infectious pathogens, and are in contact with potentially injurious antigens (Janeway et al., 2001; Kaetzel, 2005).
(6) Stratification of intestinal bacteria on the luminal side of the epithelial barrier also depend on secreted immunoglobulin A (IgA). IgA specific for intestinal bacteria is produced with the help of intestinal dendritic cells that sample the small numbers of bacteria penetrating the overlying epithelium. Some meningeal pathogens produce proteases that cleave to human immunoglobulin subclasses (e.g., IgA1), allowing adherence of bacterial strains to mucosal surfaces and crossing the mucosal barrier (Lorenzen et al., 1999; Hooper et al., 2012; Brooks and Mias, 2018). IgA1 proteases separate the pathogen-recognition (Fab) and host signaling (Fc) components of the antibody, thereby severing communication with host defense cells. This also leaves pathogens coated with cleaved Fab fragments and camouflaged from the immune system. IgA1 proteases disable this important defense immune molecule allowing for direct escape of the invading pathogen from host immunity (Woof and Russell, 2011; Marshall et al., 2017). This communication/crosstalk involving the gut microbiota from the CNS encompasses several channels along various neural, enteric and immune systems. Sensory and motor fibers from the vagus nerve connect the gut and the brainstem, and serve as a conduit for neural signals involving the microglia. Increased CNS inflammation signals vagal efferent nerves to relay information about the immune status of the brain to the gut and the gut microbes. In the same manner, vagal afferents transduce and relay information from the GIT to the CNS, signaling microglia via increased production of various pro-inflammatory cytokines that modulate neuroinflammation (Goehler et al., 1999, 2005; Borovikova et al., 2000; Forsythe et al., 2014; Abdel-Haq et al., 2019).

## Urine Reflects Dysbiosis Within Bacterial CNS Infection(S)

The CNS can communicate with the gut via signaling molecules carried by the CSF and blood, which in turn may alter gut composition and physiology. Evidence for this communication between the gut and the brain includes the following: (1) it is well known that toxins or abnormal metabolites that enter the bloodstream are ultimately removed from the blood, in an attempt to maintain a state of cellular homeostasis, and excreted via the urine (Li, 2015; Wu and Gao, 2015); (2) biomarkers for various neurological diseases are detected using body fluids including CSF, blood and urine (An and Gao, 2015). The CSF transfers waste products to the blood, which is filtered by the kidneys, whereby blood-borne waste products accumulate in the urine and are then excreted (Wu and Gao, 2015). It is also well known that various perturbations or other physiological changes in the human body – such as an altered microbiome, for instance – may change what is considered a normal urinary metabolome fingerprint into a new disease-specific fingerprint (Want et al., 2010; Emwas et al., 2015; Wu and Gao, 2015). There exists well-described examples in the literature of metabolites found in urine that are associated with microbial metabolism or microbial–host co-metabolism and found to change in response to diseases where gut dysbiosis is the predominant perturbation (Holmes et al., 2011; Vernocchi et al., 2016; Dumas et al., 2017; Malatji et al., 2019). Furthermore, urine is considered the preferred sample matrix for the detection of certain metabolites, which are otherwise difficult to detect from a blood sample due to their low concentrations. Moreover, urine collection is considered relatively non-invasive (Bouatra et al., 2013; Li, 2015). For these reasons, the metabolomics of urine has been successfully exploited for new biomarker discovery in various diseases, including neuropsychiatric disorders, such as schizophrenia, major depressive disorder, bipolar disorder, and autism spectrum disorder (Yap et al., 2010; Cai et al., 2012; Zheng et al., 2013; Chen et al., 2014), and various neurodegenerative diseases, such as PD, AD, and MS (Luan et al., 2014). Based on the premise that the urine contains the accumulation of all end-product metabolites of the body, logic dictates that chronic bacterial infection(s) of the CNS should, in principle, result in persistent feedback on the gut via the BGA, communicated via the CSF and blood, leading to dysbiosis and an altered urinary metabolome.

**Box 1. Tuberculous meningitis (TBM).**

TBM, a severe infectious disease caused by Mtb, is a chronic form of bacterial meningitis (BM), resulting in chronic neuroinflammation often associated with irreversible neurological damage/dysfunction. TBM develops in severity in progressive stages (TBM stages I, II and III), and a uniform case definition (definite, probable and possible TBM) for diagnosis has been standardized (Marais et al., 2010). TBM is the most common form of CNS-tuberculosis (TB) (Van Well et al., 2009) and is considered severe due to its high associated prevalence of mortality and morbidity (Rohlwink et al., 2019). Transmitted via infectious aerosols into the lung, Mtb may enter the circulatory system, traverse the BBB and then enter the brain meninges (Rock et al., 2008; Nicholas et al., 2012). Microglia, the resident macrophages of the brain, are the cells preferentially infected by the Mtb bacilli (Rock et al., 2005). The Rich foci (Rich and McCordock, 1933), lesions that form in the meninges, eventually rupture, spilling the Mtb microbes, cytokines and chemokines into the subarachnoid space, resulting in infection and extensive inflammation of the meninges (Dastur et al., 1995; Donald et al., 2005; Rock et al., 2008). The pathogenesis of TBM is dynamic and Mtb bacteria exhibit a resilience that allows them to survive hostile environments, which results in a persistent neuroinflammatory response if not treated correctly and swiftly (de Carvalho et al., 2010; Beste et al., 2011, 2013; Warner, 2015). Despite all efforts toward improved solutions to curbing TB since the discovery of Mtb as the causative agent in 1882, there is still a very limited understanding of Mtb infection within the host, especially so for TBM, and hence the need for new biomarkers better describing this.

In research on infectious diseases, urinary profiling has received much attention, in particular regarding pulmonary tuberculosis (TB) – a disease caused by *Mycobacterium tuberculosis* (Mtb) – about which several studies have been conducted using urine for the detection of clinically relevant biomarkers (Banday et al., 2011; Bonkat, 2012; Das et al., 2015; Luies and Loots, 2016; Luies et al., 2017; Preez et al., 2017; Isa et al., 2018). The detection of lipoarabinomannan (LAM), for instance, a *Mycobacterium*-specific liposaccharide from the Mtb cell wall, is an example of the basis of a well-studied commercial ELISA assay that shows promise for its diagnostic use in urine with a reported sensitivity of 74% and specificity of 86.9% in a study performed on 148 confirmed TB patients (Tessema et al., 2001); a sensitivity of 80.3% and specificity of 99% in a study conducted on 132 confirmed TB patients (Boehme et al., 2005); and a sensitivity of 44% and specificity of 89% in a study conducted on 195 TB-positive patients in a high-HIV prevalence setting (Mutetwa et al., 2009). Within TBM cases (see Box 1), the direct LAM-ELISA assay of CSF has similarly shown a sensitivity of 64% and specificity of 86.9% in a study including 50 TBM cases in a high-HIV-prevalence setting (Patel et al., 2009); and a sensitivity of 43% and specificity of 91% for definite TBM cases in a study performed on CSF collected from the 4th ventricle, post-mortem (Cox et al., 2015). However, Bahr et al. (2015) determined that this LAM-based TB antigen test yielded negative results for all the CSF samples (∼100) analyzed in their study, of whom 18 had a confirmed diagnosis of TBM. In a short communication the following year, Bahr et al. (2016) voiced their concern about the reliability of the LAM assay for use on CSF for diagnosis of TBM, and also discussed the study by Cox et al. (2015). Ultimately, the LAM-ELISA, like many other TB diagnostic tests, is not sufficient as a stand-alone assay for a definitive diagnosis of TB.

Of particular interest, as it pertains to our review, is that bacterial antigen-specific assays perform particularly poorly when used for diagnosing bacterial CNS infection from urine collected from patients, even in documented septicemia cases (Barnes et al., 1998). Barnes et al. postulated that the reason for this is that these complex polysaccharide antigens break down before excretion in urine. Using the well-tested LAM-ELISA assay, Blok et al. (2014) analyzed urine collected from 21 TBM cases and obtained a sensitivity of only 4.8% and specificity of 93.1%, and hence concluded that urinary LAM detection offers little value for the diagnosis of TBM. Although LAM is detectable in the urine of TB cases and the CSF of TBM patients, it is almost undetectable in urine collected from patients with TBM. A postulated reason for this inconsistency is the inability of LAM to transgress the BBB. This hypothesis can likely be extended to complex bacterial antigens in general, as supported by the results of Barnes et al. (1998). We therefore conclude from these Mtb-antigen-specific assay studies that the diagnosis of bacterial infection(s) of the CNS, based on the detection of bacterial antigens in urine, is not a viable option.

For this reason, we believe that the detection of the catabolic components (metabolites) of complex signaling pathways is a better option for the accurate and sensitive differential diagnosis of bacterial CNS infection(s), using urine collected from patients. Mason et al. (2016) provided proof-of-concept by using an untargeted gas chromatography–mass spectrometry (GC-MS) metabolomics approach to analyze the urine of 12 confirmed TBM cases, 19 non-TBM cases (sick controls proven negative for both TB and meningitis) and 29 controls. This explorative study identified urinary metabolite markers that showed two important changes in the TBM cases: (1) a dysfunctional host metabolism, and (2) indicators of an altered host–microbe response in TBM (Mason et al., 2016). The indicators of dysfunctional host metabolism included: lipolysis and ketosis (elevated 2-hydroxybutyric acid, 3-hydroxybutyric acid, 2-methyl-3-hydroxybutyric acid, and acetoacetic acid); perturbed energy metabolism (elevated branched-chain amino acid derivatives, citric acid cycle intermediates and vanillylmandelic acid); liver damage (from the presence of 4-hydroxyphenyllactic acid and 4-hydroxyphenylacetic acid, and highly elevated 4-hydroxyphenylpyruvic acid). Of greater importance to this review was the discovery of those markers serving as indicators of an altered host–microbe response in TBM, as is discussed in greater detail below.

First, Mtb-induced changes to tryptophan metabolism was evident, due to the presence of elevated urinary concentrations of indole-3-acetic acid, 5-hydroxyindole acetic acid, tryptophan, kynurenic acid and quinolinic acid, accompanied by significantly elevated levels of N-acetylanthranilic acid (the N-acetylated product of anthranilic acid; Paul and Ratledge, 1970, 1971, 1973), the latter of which is a novel microbial metabolite indicative of gut microbiota involved in the perturbed host’s tryptophan metabolism (Mason et al., 2016). Using a similar but more sensitive metabolomics analytical platform (GC × GC–TOFMS), Luies and Loots (2016) independently compared urine collected from 46 confirmed TB adults to 30 TB-negative healthy controls, and identified similar urinary markers indicative of the same alterations for the host’s tryptophan metabolism. They attributed these to the result of an inflammatory response due to releases of cytokines, specifically IFN-γ. Hence, an inflammatory response induced by Mtb-infection, whether in the lungs or brain, results in the release of IFN-γ, which stimulates the upregulation of tryptophan catabolism (Yoshida et al., 1981; Taylor and Feng, 1991; Blumenthal et al., 2012; Hashioka et al., 2017; Lu et al., 2017). The presence of increased urinary tryptophan catabolites therefore contributes to a differential diagnosis of Mtb-based infection, but they do not serve as uniquely distinctive biomarkers.

Second, Mtb–host related metabolites were identified. In particular, significantly elevated concentrations of methylcitric acid were speculated to be likely to have originated from the well-characterized methylcitrate cycle of Mtb (Muñoz-Elías et al., 2006; Savvi et al., 2008). Interestingly, a positive correlation between urinary quinolinic acid and methylcitric acid concentrations was observed by Mason et al. (2016) in all the TBM patients’ urine samples collected both before and after Mtb-specific treatment commenced. Hence, the roles of quinolinic acid and methylcitric acid in the host are intertwined during Mtb infection, and its treatment.

Lastly, urinary metabolite markers associated with alterations to the gut-microbiome were identified as a major consequence of perturbed metabolism associated with TBM. Of the significant urinary metabolites, those that are linked to gut microbiota were identified as uracil, hippuric acid, 4-hydroxyhippuric acid, phenylacetylglutamine and 4-cresol (Mason et al., 2016). Luies and Loots (2016) also identified elevated urinary concentrations of oxalic acid and rhamnulose, as evidence for an altered gut-microbiome in pulmonary TB. In a follow-up study by Luies et al. (2017), the failure of treatment of TB via standard anti-TB combination therapy was characterized by an imbalanced gut-microbiome, with the two largest predictors for a poor treatment outcome being two altered micobiome urinary markers [3,5 dihydroxybenzoic acid and 3-(4-hydroxy-3-methoxyphenyl)propionic acid]. Additionally, another independent GC-MS metabolomics longitudinal treatment study conducted on TB patient urine (Das et al., 2015) showed a treatment-dependent trend of a deregulated tyrosine–phenylalanine axis, also associated with an abnormal microbiome. Considering these urinary TB metabolomics studies, although not yet fully understood, strong evidence exists for the association of TB disease and an altered microbiome, detectable via altered metabolite markers present in urine collected from TB patients.

Independent urinary metabolomics studies on pulmonary TB, therefore, although not related to the CNS but still involving an infectious disease distinguished by chronic inflammatory response(s), support the findings of Mason et al. (2016) in characterizing chronic neuroinflammation from TBM through urinary profiling. Herein lies the strength of untargeted metabolomics studies – the complementary evidence of three independent, open-minded analyses of metabolomics data obtained from urine on a similar analytical platform with a common, general hypothesis of the importance of the gut microbiota. For the remainder of this review, we focus on TBM and take a validated 3-marker CSF immunological signature of TBM and discuss it in conjunction with previously identified, altered urinary metabolomics markers of TBM.

## Validated 3-Marker CSF Immunological Signature of TBM

Bacteriological confirmation of TBM from CSF is not always possible, especially in children, so that diagnosis is mostly based on a combination of clinical findings, CSF analysis and radiological results (Marais et al., 2010). Since various biomarker-based tests of the host have shown promise in extrapulmonary pleural-TB diagnostics, it has been thought that these same tests could also be used to diagnose TBM (Chegou et al., 2008). Recent technology has allowed for the screening for many such biomarkers, using as little as 3 μL of CSF via Luminex multiplex cytokine-beaded arrays. With clinical application, host biomarkers could potentially be added to the current TBM diagnostic armamentarium, in order to provide an earlier and more efficient diagnosis.

A preliminary 3-marker CSF biosignature, comprising VEGF, IL-13 and cathelicidin LL-37 (cut-off values 42.92, 37.26, and 3221.01 pg/mL, respectively), correctly diagnosed childhood TBM with a sensitivity and specificity of 52 and 95%, respectively (Visser et al., 2015). The same 3-marker CSF biosignature, tested on a different cohort of 23 children, however, revealed lower sensitivity (30.4%), yet a similar specificity (91.7%), with different cut-off values. In this same cohort of 23 children with TBM and 24 controls, VEGF, IFN-γ, and MPO provided good accuracy with an AUC of 0.97, up to 91.3% sensitivity and up to 100% specificity, with cut-off values of >9.4, >99.5, and >25,823 pg/mL, respectively (Manyelo et al., 2019). Hence, VEGF, IFN-γ, and MPO in combinaton was validated by Manyelo et al. (2019) as a 3-marker CSF immunological signature of TBM. The background behind these three markers is now described, in order to provide insights into how they led to our predictive metabolic model.

## Vascular Endothelial Growth Factor (VEGF)

VEGF, a 46 kDa glycosylated homodimeric cytokine protein, is expressed intracellularly in several cell types, including microglia (Cohen et al., 1996). It is a potent growth factor inducer of vascular endothelial cell proliferation, vascular permeability (Soker et al., 1997) and angiogenesis (Connolly, 1991; Yancopoulos et al., 2000). Endothelial changes associated with VEGF include: (1) separation of intercellular tight junction, (2) increased vesicle transport, and (3) formation of vesico-vacuolar organelles, all of which results in increased macromolecular transport over the endothelial barrier (Feng et al., 1996; Wang et al., 2001). Classically associated with chronic inflammatory diseases, such as rheumatoid arthritis (Fava et al., 1994), VEGF is also associated with the increased permeability, and subsequent dysfunction, of the BBB (Dobrogowska et al., 1998; Proescholdt et al., 1999; Harrigan et al., 2002) and in the pathogenesis of brain edema related to ischemia, trauma, vasculitis and tumors (Van Bruggen et al., 1999; Viac et al., 1999). VEGF exhibits direct neuroprotective effects during *in vitro* ischemia (Jin et al., 2000). Another study showed that topical application of VEGF on the cerebral cortex induces a reduction of infarct size in a rat model of transient cerebral ischemia (Hayashi et al., 1998).

In 2001, Van der Flier et al. showed no detectable CSF VEGF concentrations in patients with viral meningitis (VM), whereas 30% (11/37) of those patients with bacterial meningitis (BM) displayed detectably elevated concentrations of CSF VEGF (ranging from <25 to 633 pg/mL). Furthermore, elevated VEGF has been associated with an upregulation of MMP-9 (Wang and Keiser, 1998) – see Box 2 – which additionally contributes to BBB disruption in BM (Paul et al., 1998). Van der Flier et al. (2001) also indicated the VEGF index in BM (calculated as [VEGF_CSF_/VEGF_plasma_]/[albumin_CSF_/albumin_plasma_]) to be 6.2 [0.6–42], which indicates that CSF VEGF is a result of intrathecal production. This increase in CSF VEGF could be associated with: (1) a change in mental status, (2) seizures, (3) an elevated CSF WBC count (with neutrophils being the main source of VEGF), (4) elevated CSF protein and higher CSF:serum albumin ratios (marker of BBB breakdown), (5) severe BBB disruption, and, eventually, (6) death.

**Box 2. Matrix metalloproteinases (Kolb et al., 1998; Leib et al., 2000; Shapiro et al., 2003; Lee et al., 2004).**

MMPs are a large family of zinc-dependent proteolytic enzymes. Their main function involves remodeling of the connective tissues by degrading extracellular matrix molecules and are regulated by tissue inhibitors of metalloproteinases. These many compounds are subdivided according to their main substrates:

• Gelatinases: MMP-2, MMP-9.
• Collagenases: MMP-1, MMP-8, MMP-13.
• Stromelysins: MMP-3, MMP-10, MMP-11.

MMP-2 and MMP-9 digest type IV collagen and are subsequently implicated in the breakdown of the BBB via dissolution of the basement membrane underlying the endothelial cells. MMP-2 and MMP-9 production is strongly correlated with the development of neurological sequelae and induced by pro-inflammatory cytokines (IFN-γ) and other mediators (such as MPO). The amount of MMP present in CSF varies, depending on the severity of inflammation. MMP-2 and MMP-9 are detected in elevated amounts in the CSF of meningitis cases (TBM, VM and BM), with MMP- 9 correlating strongly with the number of neutrophils in VM.

Within TBM, VEGF is localized in the microvessels and perivascular cells (Matsuyama et al., 2001). Tumor necrosis-alpha (TNF-a), associated with pathogenesis of TBM (Tsenova et al., 1999), is a known inducer of VEGF (Ryuto et al., 1996). In a follow-up investigation conducted by Van der Flier et al. (2004), the prevalence of elevated CSF VEGF concentrations in TBM patients was 58% (15/26) (at 98 ± 31 pg/mL) with a calculated VEGF index of 486 ± 976, the latter once again indicative of intrathecal production. Van der Flier et al. furthermore associated the elevated concentrations of CSF VEGF in TBM with: (1) significantly greater mononuclear cell counts; (2) elevated CSF protein and higher CSF:serum albumin ratios; (3) not being significantly correlated with the elevated ICP, decreased CSF glucose nor with cerebral infarct on a CT scan; and (4) the inhibition explained the clinical effect of adjuvant corticosteroid therapy. In 2008, Hussain et al. similarly indicated significantly increased CSF VEGF levels (106 ± 50 pg/mL [44.9–336 pg/mL]) in TBM, accompanied by a strongly positive correlation between microvessel density and VEGF expression. Additionally, the investigation revealed that in excised tuberculomas: (1) VEGF expression was highest in regions of the granulomatous reaction; (2) no VEGF was present in the areas of caseous necrosis; (3) areas of caseation were devoid of angiogenesis; and (4) inflammatory mononuclear cells were positive for VEGF antigen (these included epitheloid cells, histiocytes and macrophages). Furthermore, immunohistochemical staining of excised tuberculoma demonstrated an elevated expression of VEGF in the granulomatous areas, with positivity in inflammatory mononuclear cells, Langhan’s giant cells, as well as reactive astrocytes and fibrocytes.

Matsuyama et al. (2001) and Visser et al. (2015) both indicated CSF VEGF to be significantly increased in TBM compared with other types of meningitis (Table 2). Among the TBM cases, CSF VEGF was additionally significantly higher in those patients with hydrocephalus (196.3 ± 60.2 pg/mL vs. 119.8 ± 69.6 pg/mL) and there was a significant correlation with increased CSF protein and CSF total cell counts (Matsuyama et al., 2001). Visser et al. (2015) associated elevated CSF VEGF with raised hydrocephalus and CSF protein (>1 g/L), along with basal meningeal enhancement and hyperdensity in the basal cisterns on non-contrast CT scans. Lastly, Matsuyama et al. (2001) indicated that CSF VEGF localizes to microvessels and perivascular cells in TBM.

**TABLE 2:** Summary of CSF VEGF concentrations in different types of meningitis.

	**TBM**	**BM**	**VM**
CSF VEGF	142.8 pg/mL [28.1–225.7]^a^ 144.4 ± 75.1 pg/mL^d^ 106 ± 50 pg/mL [44.9–336]^e^	14.5 pg/mL [8.7–86.5]^a^ 47 ± 9 pg/mL [<10–174]^b^ 37.5 pg/mL [<20–160]^c^ 80.1 ± 49.5 pg/mL^d^	27.9 pg/mL [7.9–48.7]^a^ 27.6 ± 26.3 pg/mL^d^

*^*a*^Visser et al. (2015). ^*b*^Van der Flier et al. (2005). ^*c*^Coenjaerts et al. (2004). ^*d*^Matsuyama et al. (2001). ^*e*^Husain et al. (2008).*

## Myeloperoxidase

Myeloperoxidase (MPO), a heme enzyme (EC 1.11.1.7) and pro-inflammatory mediator present in the primary granules of polymorphonuclear leukocytes (PMNs), participates in oxygen-dependent microbiocidal activity of PMNs and triggers oxidative stress during acute and chronic inflammatory processes, resulting in the production of ROS. MPO can be measured in CSF as an index of inflammation (Liechti et al., 2014) and leukocyte influx (Grandgirard et al., 2012). In a review by Ray and Katyal (2016), MPO was clearly associated with the etiology of neurodegenerative disorders.

MPO is synthesized in reaction to infection (Pohanka, 2013), resulting in elevated ROS. The occurrence of oxidative stress in meningitis patients is well-described in the literature (Koedel and Pfister, 1999; Ray et al., 2000; Tsukahara et al., 2000; Christen et al., 2001; Kastenbauer et al., 2002; Klein et al., 2006; Hamed et al., 2009; Loro, 2009; Koedel et al., 2010; Mirić et al., 2010; Barichello et al., 2011). Furthermore, significant increases in MPO activity have been shown in BM-induced rats (Giridharan et al., 2017), particularly within the hippocampus and frontal cortex (Barichello et al., 2011, 2014). In a study of 59 pediatric BM cases, Mirić et al. (2010) showed no significant correlation between MPO and neutrophil count in CSF; however, CSF MPO activity did correlate with various lipid peroxidation products. Additionally, H_2_O_2_ levels in CSF were associated with elevated BBB permeability, CSF albumin concentrations, and serum H_2_O_2_ concentrations. Lastly, it is important to note that MPO reacts with cell matrix metalloproteinases (MMPs – see Box 2), or their tissue inhibitors, and this is thought to contribute to the BBB dysfunction seen in such cases.

Borelli et al. (1999) proved that purified MPO, in the presence of H_2_O_2_, exerts a consistent killing effect on Mtb, and that the MPO activity is both time and dose dependent; it also requires chloride ions for efficacy. This MPO–H_2_O_2_–Cl_2_ system produces hypochlorous acid (HOCl) via activated leukocytes (Klebanoff, 2005), which in turn serves as a strong, non-radical oxidant of a wide range of biological compounds, although it is more selective than hydroxyl radicals (Hampton et al., 1998), with the following characteristics: (1) it has a preferred substrate selectivity toward thiols and thioethers, (2) an ability to convert amines to chloramines, (3) promotes chlorination of phenols and unsaturated bonds, (4) oxidizes iron centers, (5) crosslinks proteins, and (6) is membrane permeable. HOCl has also been characterized as covalently modifying lipids and/or proteins, resulting in local tissue damage and amplification of the inflammatory cascade. Furthermore, HOCl, in the presence of nitrite (NO^2–^) formed by stimulated PMNs, forms 3-chlorotyrosine (3Cl-Tyr), and to a lesser degree, 3-nitrotyrosine (3NO_2_-Tyr) and N-chlorotaurine (Eiserich et al., 1998). The 3Cl-Tyr is considered a specific marker of MPO-catalyzed oxidation (Hazen and Heinecke, 1997), with GC-MS being the preferred method for quantifying it (Hazen et al., 1997; Winterbourn and Kettle, 2000). Other biomarkers of MPO-derived HOCl include: chlorohydrins, protein carbonyls, anti-HOP (hypochlorous acid-oxidized protein), antibodies, 5-chlorocytosine, and glutathione sulfonamide. Each with their advantages and disadvantages is described by Winterbourn and Kettle (2000). Based on the analyses of CSF collected from 79 confirmed pediatric BM cases, Rugemalira et al. (2019) indicated that elevated ratios of 3Cl-Tyr:para-tyrosine serves as a marker for MPO activation in CSF in pediatric BM cases, and potentially also for grading the severity of neuroinflammation. Furthermore, Rugemalira et al. (2019) also proved that 3NO_2_-Tyr can be used as a biomarker for peroxynitrite formation and is associated with an unfavorable outcome of BM. In a study of 59 children with confirmed BM (Mirić et al., 2010), CSF MPO activity, although relatively low, was significantly increased at baseline compared to controls (*n* = 23), increasing even further by day 5 of treatment. It was concluded that MPO may be involved in the oxidative stress associated with BM, as well as potentially contributing to BBB disruption. Marais et al. (2016) indicated a significant increase in neutrophil-dependent inflammatory response biomarkers, including MPO, in adult TBM and HIV co-infection patients with paradoxical immune reconstitution inflammatory syndrome. Lastly, Üllen et al. (2013) indicated that BBB dysfunction associated with neuroinflammation caused by MPO can be partially reversed by using para-aminobenzoic acid (PABA) hydrazide, first shown by Forghani et al. (2012) to effectively treat multiple sclerosis in mice. PABA (or vitamin Bx) is non-essential for humans, but exhibits anti-fibrotic properties. Fibrosis in the brain occurs via the proliferation or hypertrophy of glial cells, such as microglia – microgliosis, during neurotrauma caused by infection. Subsequently, PABA may later be considered for its use as a possible adjunctive therapeutic agent in TBM, since the inhibition of MPO has been posited to be a valuable therapeutic approach to reduce oxidative-stress-mediated damage in neurodegenerative diseases (Green et al., 2004).

## Interferon-γ

Interferon-γ (IFN-γ) is predominantly produced by CD4^+^ T cells and functions by activating microglia, thereby stimulating lymphocyte Th1 differentiation (Farrar and Schreiber, 1993) and antimicrobial activity of the microglia (Mastroianni et al., 1997), after infection. A plethora of literature studies report the performance of IFN-γ release assays (IGRAs) for diagnosing TB under different conditions. These studies are comprehensively covered by systematic reviews and meta-analyses and include applications to diagnosing: (1) latent Mtb infection (53 studies: Diel et al., 2011); (2) latent Mtb infection in rheumatic patients (11 studies: Ruan et al., 2016); (3) latent TB in patients with autoimmune diseases under immunosuppressive therapy (17 studies: Wong et al., 2016); (4) active TB (27 studies: Sester et al., 2011); (5) active TB among HIV-seropositive individuals (11 studies: Huo and Peng, 2016); (6) active TB in immunocompetent children (15 studies: Laurenti et al., 2016), immunodiagnosis of TB (75 studies: Pai et al., 2004); (7) active and latent TB in HIV-positive populations (32 studies; Overton et al., 2018); and (8) extra-pulmonary TB (22 studies: Zhou et al., 2015). Similarly, several studies (Table 3) using IGRAs have also been performed using CSF as a possible sample matrix for diagnosing TBM, with the two main commercially used IGRAs tested being T-SPOT. TB and QuantiFERON-TB. IGRAs function by measuring the release of IFN-γ from T cells, after *in vitro* stimulation with Mtb antigens, such as early secreted antigenic target 6 (ESAT-6) and culture filtrate protein 10 (CFP-10); they are influenced by (1) the antigenic load, (2) host responsiveness to antigens, and (3) host–pathogen interactions (Lu et al., 2017).

**TABLE 3:** Performance of IGRAs on CSF from TBM cases as a stand-alone diagnostic tool.

**References**	**IGRA**	**TBM cases (n)**	**Sensitivity % (range)**	**Specificity % (range)**
Pan et al. (2017)	T–SPOT.TB	53	61(40–92)	97(75–100)
Lu et al. (2017)	T–SPOT.TB	61	62(49–74)	73(62–82)
Qin et al. (2015)	T–SPOT.TB	12	92(62–100)	93(76–99)
Park et al. (2012)	T–SPOT.TB	25	72(51–88)	79(66–89)
Kim et al. (2010)	T–SPOT.TB	31	71(51–86)	89(72–98)
Patel et al. (2010)	T–SPOT.TB	38	58(41–74)	94(83–99)
Thomas et al. (2008)	T–SPOT.TB	10	90(56–100)	100(59–100)
Caliman-Sturdza et al. (2015)	QuantiFERON-TB	63	84	98
Vidhate et al. (2011)	QuantiFERON-TB	36	13(2–40)	63(35–85)
Weighted average diagnostic performance of IGRAs		329	65	87

Consolidating from the literature, the CSF studies on IGRAs as a diagnostic tool for TBM (Table 3), a weighted average of the diagnostic performance of IGRAs (pooled from 326 TBM cases) was calculated to give an overall average sensitivity and specificity of 65 and 87%, respectively – insufficient for application as a stand-alone diagnostic tool. On similar data, a meta-analysis of 6 studies from the literature, all using IGRAs conducted on CSF, showed a pooled (156 cases) sensitivity of 77% (69–84%) and specificity of 88% (74–95%) for TBM diagnostic applications (Yu et al., 2016). Furthermore, IGRAs require 3–7 mL of CSF, a volume often unobtainable, especially from children and infants. Moreover, the measure of sensitivity and specificity is dependent upon a pre-defined cut-off point which is currently not yet standardized.

The use of IGRAs for the differential diagnosis of meningitis has, however, yielded a practical outcome. Chonmaitree and Baron (1991) analyzed CSF from 16 VM and 41 BM cases and determined that elevated concentrations of IFN-γ were present in 75 and 24% of these patient groups, respectively. A review of the literature (1964–1991) by Chonmaitree and Baron (1991) revealed a similar trend, showing elevated concentrations of IFN-γ in 68% (133/196) of all VM patients (based on 11 studies), whereas in patients with BM, only 28% (59/189) showed elevated IFN-γ in the pooled population (8 studies used). Hence, patients with VM exhibit higher IFN-γ levels than those with BM. Based upon quantified data in 50 patients with VM, using a radioimmunoassay, Minamishima et al. (1991) determined CSF IFN- γ to be on average 9.8 ± 7.5 UI/mL. Minamishima et al. additionally suggested that IFN-γ produced in the inflamed intrathecal space may be associated with the pathogenesis of the disease, and associated the elevated CSF IFN-γ levels with (1) CSF protein concentrations, (2) total cell counts, and (3) number of febrile episodes. San Juan et al. (2006), also using a radioimmunoassay on CSF collected from patients, calculated a mean IFN-γ for definite (*n* = 12) and probable (*n* = 8) TBM patients to be 28.7 ± 8.2 and 10.6 ± 2.8 UI/L, respectively. However, Ohga et al. (1994) showed only 3 out of the 13 BM patients investigated, and Kornelisse et al. (1997) only 20 of 35 BM patients investigated, to have CSF IFN-γ elevated to concentrations above the detection limit of 10 pg/mL. In an analysis of 30 TBM patients, Lu et al. (2016) determined, via ELISA, a mean CSF IFN-γ value for patients with TBM to be 350.97 ± 372.94 pg/mL. Lu et al. also determined that in 10 of these TBM patients the average CSF IFN-γ levels were 500.48 pg/mL before treatment and 103.62 pg/mL following 4 weeks of treatment, indicating that while IFN-γ decreased significantly (5-fold), it still remained elevated compared to the norm, after 4 weeks of treatment (that is, inflammation in the brain persisted). Mansour et al. (2005) reported a highly elevated mean concentration of CSF IFN-γ (794 ± 530 pg/mL) in 39 patients with TBM (all of whom were HIV negative) prior to receiving medication, which was correlated with markers of neuroinflamation in these individuals. Mansour et al. (2005) also showed that the CSF IFN-γ remained elevated for many weeks after treatment was begun in patients with TBM, whereas in those cases diagnosed with VM and BM the CSF IFN-γ returned to undetectable concentrations within a couple of days post-treatment. Considering all of the above, patients with VM and TBM exhibit a similar increase in CSF IFN-γ levels, both far greater than in patients with BM. This suggests that CSF IFN-γ could potentially be used as a differential diagnostic marker for the exclusion of BM. Furthermore, CSF IFN-γ levels in TBM cases remain elevated for weeks following treatment, differentiating TBM from VM. However, as described previously, in order to acquire a definitive TBM diagnosis additional measures of CSF parameters are needed.

In summary, the overall trend across all CSF VEGF studies is a significantly higher concentration of VEGF in TBM patients than in other cases of meningitis. Of further note, Van der Flier et al. (2004) reports significantly increased CSF VEGF (178 ± 52 pg/mL) in TBM patients with nausea and vomiting, indicating that elevated CSF VEGF has a potential direct impact on the BGA, leading to a perturbed gut. CSF IFN-γ levels show a similar increase in TBM and VM but less so in BM. Hence, CSF IFN-γ levels could potentially be used for the exclusion of the diagnosis of BM. The HOCl produced by the MPO–H_2_O_2_–Cl_2_ system yields similar oxidative markers in both TBM and BM.

The addition of VEGF and MPO with IFN-γ, as part of a 3-marker immunological biosignature of TBM in CSF (Manyelo et al., 2019), has yielded a diagnostic measure with an AUC of 0.97, and a sensitivity and specificity of up to 91.3% and up to 100%, respectively. Hence, this 3-marker biosignature yields excellent results for diagnosis of TBM from a CSF sample. But, what of the urinary metabolomics profile? If these three immunological markers are present in the CSF of a TBM patient, then a downstream metabolic effect, based upon the BGA, should be reflected in the urine. This concept is explored in our proposed predictive metabolic model that follows.

## Proposed Predictive Metabolic Model of TBM in the Brain Based Upon IFN-γ, MPO and VEGF

**FIGURE 1:**
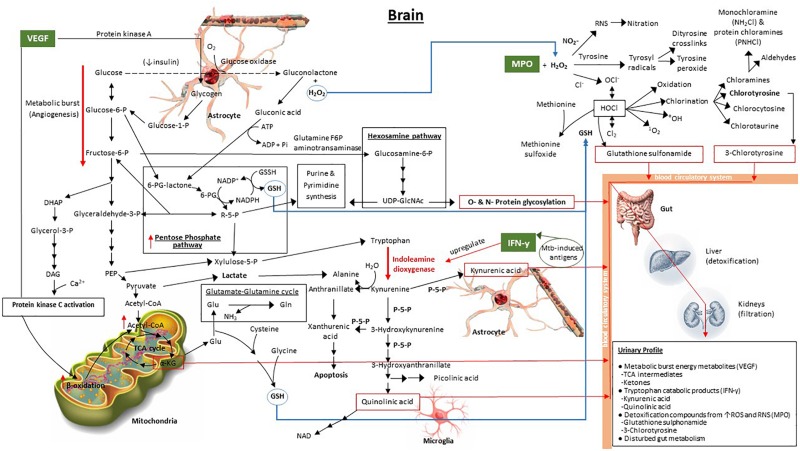
Model predicting the downstream metabolic effects of VEGF, IFN-γ, and MPO in the brain, and the expected attendant urinary profile, in a TBM patient. The bold red arrows indicate the major upregulated metabolic pathways, namely tryptophan catabolism, induced by upregulated indoleamine dioxygenase due to IFN-γ, and increased metabolic burst (angiogenesis) via increased glycolysis. The red boxes enclose metabolic end-products within the Mtb-infected brain – protein glycosylation, α-ketoglutarate, quinolinic acid, kynurenic acid, 3-chlorotyrosine and glutathione sulfonamide – which are the predicted metabolic brain markers of TBM. The blue arrows indicate the transport of important metabolic components. The dashed black line, indicating oxidation of glucose to gluconolactone, represents a transient pathway that occurs when insulin is depleted, as indicated. 6-PG, 6-phosphogluconate; R5P, ribulose-5-phosphate; UDP-GlcNAc, UDP-N-acetylglucosamine; PEP, phosphoenolpyruvate; DHAP, dihydroxyacetone phosphate; DAG, diacylglycerol; TCA cycle, tricarboxylic acid (citric acid) cycle; α-KG, α-ketoglutarate; Glu, glutamate; Gln, glutamine; GSH, glutathione (reduced); P-5-P, pyroxidal-5-phosphate (vitamin B_6_).

Given the background of IFN-γ, MPO, and VEGF described above, and the associated metabolic pathways of these signaling compounds, we propose a predictive metabolic model for TBM in the brain based upon previously published biochemistry fundamentals. This model, illustrated in Figure 1, shows the interaction of the overlapping metabolic cascades initiated by TBM, and its associated 3-marker CSF immunological signature.

Our predictive metabolic model shows how increased levels of VEGF result in a persistent metabolic burst caused by the induction of angiogenesis (Stapor et al., 2014; Treps et al., 2016), whereby glycolysis, and the release of glycogen from astrocyte stores to fuel glycolysis, is increased significantly. Secondary pathways that are subsequently upregulated include: (1) the pentose phosphate pathway, that contributes to an elevated synthesis of glutathione (Ben-Yoseph et al., 1996), elevated xylulose-5-phosphate (also via phosphoenolpyruvate in the glycolysis pathway) to fuel tryptophan catabolism (Stephanopoulos and Simpson, 1997; Simpson et al., 1999; Maria et al., 2018), and elevated purine and pyrimidine synthesis (Zimmer, 1988, 1996); (2) the hexosamine pathway, which contributes to increased O- and N-protein glycosylation, imperative for the host’s immune response since glycosylation controls cell migration, host defense, and antigenicity (Varki, 1993); (3) increased β-oxidation providing substrate in the form of diacylglycerol from downstream catabolism of dihydroxyacetone phosphate and activation of protein kinase C from VEGF (Takahashi et al., 1999; Harhaj et al., 2006), ultimately yielding increased acetyl-CoA; and (4) the boosted mitochondrial citric acid (TCA) cycle, due to the increased acetyl-CoA. The elevated TCA intermediate α-ketoglutarate (α-KG), previously indicated to be a urinary marker of TBM (Mason et al., 2016), contributes to glutamate synthesis and downstream glutathione (GSH) production, the latter being a needed antioxidant, synthesized in response to the elevated MPO.

Increased levels of IFN-γ, stimulated by Mtb-induced antigens (Blumenthal et al., 2012; Lu et al., 2017), specifically upregulate indoleamine dioxygenase (Yoshida et al., 1981; Taylor and Feng, 1991; Hashioka et al., 2017), the initial enzyme in the tryptophan catabolic pathway. A massive burst in tryptophan catabolism results in astrocyte-based kynurenic acid and microglia-based quinolinic acid synthesis – also previously identified urinary markers of TBM (Mason et al., 2016). Several enzymes within the tryptophan metabolic pathway require pyridoxal-5-phosphate (P-5-P), an active form of vitamin B_6_, as a cofactor. Deficiency of P-5-P diverts tryptophan metabolism from production of NAD to the excessive formation of xanthurenic acid (Oxenkrug, 2013), and subsequently apoptosis. One of the mechanisms of insulin resistance is inflammation-induced upregulation of tryptophan metabolism in combination with P-5-P-deficiency-induced diversion of tryptophan metabolism leading to formation of xanthurenic acid and other kynurenine derivatives that affect insulin activity (Oxenkrug, 2013). It has been shown that reduced insulin levels may lead to uncontrolled glucose metabolism – previously described in individuals with diabetes (American Diabetes Association, 2013), pulmonary TB (Preez et al., 2017) and, recently, in runners after a marathon (Stander et al., 2018), all of which are associated with a severe inflammatory response. As depicted in our model, uncontrolled glucose metabolism can result in glucose being oxidized via glucose oxidase to produce gluconolactone, in addition to hydrogen peroxide, previously reported to occur in both diabetes and pulmonary TB (Preez et al., 2017). The gluconolactone subsequently becomes siphoned into the pentose phosphate pathway via hydrolysis to form gluconic acid and phosphorylation with ATP to produce 6-phosphogluconate lactone (Dickens and Glock, 1951; Rohatgi et al., 2014). The consequential elevated H_2_O_2_, and its interaction with raised MPO, leads to the activation of various oxidative stress pathways (Hampton et al., 1998; Podrez et al., 2000; Klebanoff, 2005), as depicted in Figure 1, and described above. The two final urinary markers of elevated MPO and of HOCl, via the MPO–H_2_O_2_–Cl_2_ system, are predicted to be glutathione sulfonamide and 3-chlorotyrosine (Winterbourn and Kettle, 2000).

Furthermore, the burst from both the glycolysis (via pyruvate) and tryptophan catabolism (via hydration of kynurenine to form alanine; Kotake and Nakayama, 1941) pathways yields increasingly elevated levels of extracellular lactate. This lactate pool is essential in the lately proposed astrocyte–microglia lactate shuttle (AMLS, Mason et al., 2015), since lactate plays a dual role of being a preferred source of energy in the brain during TBM as well as also being neuroprotective (Mason, 2017), diverting lactate away from neurons (thereby deactivating neurons to protect them) toward activated microglia in an attempt to eradicate the immediate insult/infection. This increase in lactate in TBM is predicted to be localized in the brain for immediate use and is not expected to be present in elevated amounts in the urine of TBM patients.

The predictive metabolic model presented here, although speculative, is based upon three validated immunological markers of TBM. The subsequent activated metabolic pathways in the brain are based upon biochemistry fundamentals and supported by the literature, as discussed. The end-product metabolites that act as metabolic markers of TBM are expected to cross the BBB and travel in the blood circulation and interact with the gut. The principal limitation of our model is that, in its current form, it can predict only the end-product metabolites from the TBM-infected brain, with the assumption that no other systemic co-infection is present. The complex interactions with the gut microbiota are poorly understood and require further research. However, based upon previous urinary metabolomics studies reported in this review, experimental evidence is emerging that points toward an altered gut metabolism. Hence, changes in gut metabolism became the fourth component of our proposed urinary metabolic profile of a TBM patient. The specifics of the complex relationship between host and gut-microbiome and the details of the altered metabolic profile of the gut under pathophysiological states remain a hot topic.

## Conclusion

The significance of this review is that it takes a newly established paradigm within the neurosciences – the BGA – and critically examines the literature from a relatively unexplored niche perspective – chronic neuroinflammation caused by CNS bacterial infection(s) of the brain, using TBM as an example. We posit that if the BGA exists then chronic neuroinflammation within the brain caused by pathogenic bacteria (Mtb) will influence the gut microbiota, and the ideal biofluid to analyze this, reflecting the associated systemic changes, is urine. We support our postulate with data from published studies on urinary metabolomics, as follows.

First, the strength of untargeted urinary metabolomics is clearly demonstrated in the literature. A previous untargeted urinary metabolomics study conducted on TBM cases, by Mason et al. (2016), yielded data that, when analyzed in a non-biased, holisitic manner, resulted in a putative urinary metabolic signature characterizing TBM that was interpreted in a hypothesis-generating perspective. Independently, using similar analytical platforms in metabolomics, Das et al. (2015) and Luies and Loots (2016) examined urinary metabolomics profiles of pulmonary TB patients, and came to similar conclusions – the most significant of which, in the context of this review, were that infection by Mtb results in an altered gut-microbiome and this is substantiated by altered microbiome markers in the urine of these patients.

Second, we take an independent, and initially unrelated, study that closely examined the immunological profile of TBM, in which three specific immunological markers in the CSF associated with neuroinflammation – VEGF, IFN-y, and MPO – were validated as diagnostic markers of TBM. We explored the background behind this 3-marker CSF immunological signature of TBM, in the context of its influence on the gut-microbiome and the subsequently altered urinary metabolome, using previously discovered urinary metabolites in TBM patients as proof (such as α-KG, and the tryptophan catabolites 3-hydroxykynurenic acid and quinolinic acid) (Mason et al., 2016). By extension, we also predict other metabolic pathways that would be expected to be changed within our model.

Third, we combined the sciences of immunology and metabolomics to create a novel integrated predictive metabolic model of TBM in the brain. By integrating relevant information from systems biology, our predictive cascading metabolic model should be adjustable to account for other types of bacterial infection(s) of the CNS that cause chronic neuroinflammation, such as neurosyphilis, bacterial brain abscesses and Lyme disease, as well as chronic non-bacterial CNS infections that are common in resource-constrained settings of poor communities, and sometimes difficult to distinguish from TBM, such as cerebral malaria and cryptococcal meningitis. Being so identified, based upon the literature, patients with VM and TBM exhibit a similar increase in CSF IFN-γ levels, both far greater than in patients with BM. Hence, a predictive metabolic model of cerebral malaria and cryptococcal meningitis would likely exclude CSF-based IFN-y and its subsequent downstream cascading metabolic influence – that is, no downstream tryptophan metabolic catabolites. What remains to be done is to identify the unique immunological markers associated with these other bacterial infection(s) of the CNS and predict and confirm their associated downstream metabolic markers that should be reflected in urine, which could be used diagnostically or to characterize these diseases better.

In short, analysis of urinary metabolic profiles offers a wealth of metabolic information that can be traced back to an altered gut-microbiome, and to an inherently changed BGA, induced by chronic neuroinflammation from bacterial infection(s) of the CNS. This metabolic information from urine holds within it the potential to contribute to improved and early differential diagnosis of bacterial infection(s) in the CNS – a quicker and less invasive method of diagnosis than currently available. The review presented here provides support that, by taking existing validated immunological markers of infectious diseases in conjunction with metabolomics data and biochemistry fundamentals, it is possible to predict downstream metabolic products, most likely detectable via urinary metabolic profiling methods.

## Author Contributions

SM conceptualized the manuscript. SI, SM, and DL planned the outline of the manuscript. SI wrote the manuscript. SM and DL supervised SI in the writing of the manuscript by providing critical feedback. RS provided clinical input and critically read the manuscript. All co-authors read and approved the final draft for submission.

## Conflict of Interest

The authors declare that the research was conducted in the absence of any commercial or financial relationships that could be construed as a potential conflict of interest.
